# Dissecting Germ Cell Metabolism through Network Modeling

**DOI:** 10.1371/journal.pone.0137607

**Published:** 2015-09-14

**Authors:** Leanne S. Whitmore, Ping Ye

**Affiliations:** 1 School of Molecular Biosciences, Washington State University, PO Box 647520, Pullman, Washington, 99164, United States of America; 2 Department of Molecular and Experimental Medicine, Avera Cancer Institute, 1000 E 23rd Street, Sioux Falls, South Dakota, 57105, United States of America; Mayo Clinic, UNITED STATES

## Abstract

Metabolic pathways are increasingly postulated to be vital in programming cell fate, including stemness, differentiation, proliferation, and apoptosis. The commitment to meiosis is a critical fate decision for mammalian germ cells, and requires a metabolic derivative of vitamin A, retinoic acid (RA). Recent evidence showed that a pulse of RA is generated in the testis of male mice thereby triggering meiotic commitment. However, enzymes and reactions that regulate this RA pulse have yet to be identified. We developed a mouse germ cell-specific metabolic network with a curated vitamin A pathway. Using this network, we implemented flux balance analysis throughout the initial wave of spermatogenesis to elucidate important reactions and enzymes for the generation and degradation of RA. Our results indicate that primary RA sources in the germ cell include RA import from the extracellular region, release of RA from binding proteins, and metabolism of retinal to RA. Further, *in silico* knockouts of genes and reactions in the vitamin A pathway predict that deletion of *Lipe*, hormone-sensitive lipase, disrupts the RA pulse thereby causing spermatogenic defects. Examination of other metabolic pathways reveals that the citric acid cycle is the most active pathway. In addition, we discover that fatty acid synthesis/oxidation are the primary energy sources in the germ cell. In summary, this study predicts enzymes, reactions, and pathways important for germ cell commitment to meiosis. These findings enhance our understanding of the metabolic control of germ cell differentiation and will help guide future experiments to improve reproductive health.

## Introduction

Continuous production of sperm throughout reproductive life is a fundamental feature of mammalian spermatogenesis. Understanding the complex process of spermatogenesis has important implications for male infertility and contraception. Presently, 70 million (i.e., 15%) married couples worldwide are infertile and half of these cases are attributed to male factors [1]. In approximately 25% of male infertility cases, the cause is unknown and treatment options are limited. Additionally, men have fewer options for contraception compared to women. There is a clear need to further our understanding of male reproduction in order to address infertility and contraceptive issues.

The initial development of spermatogonia to spermatozoa is termed as the first wave of spermatogenesis. It commences prior to puberty and is followed by subsequent rounds of spermatogenesis. Retinoic acid (RA), a derivative of vitamin A, is required for several processes during spermatogenesis, including the transition from undifferentiated to differentiating spermatogonia [2–4]. This differentiation event commits germ cells to meiosis and subsequently the formation of mature spermatozoa. Recent evidence showed that the highest levels of RA within the testis were detected when spermatogonial differentiation took place, both in neonatal and adult mice [5]. The pulse of RA represents a sharp increase in RA concentration every 8.6 days in the testis. This observation suggests that pulses of RA are likely the stimulus for both the initial wave and subsequent rounds of spermatogenesis.

It remains unclear which enzymes and reactions in which cell types synthesize or degrade RA, thereby generating the RA pulse. Evidence suggests that RA from Sertoli cells—the somatic supporting cells—is critical for the first wave of spermatogenesis but not for subsequent waves [6, 7]. However, the role of germ cells in regulating RA pulse has not been thoroughly investigated. There is a crucial need to better understand whether or how vitamin A metabolism in the germ cell contributes to the pulse of RA.

Other metabolic pathways may be crucial in regulating spermatogonial differentiation through connection with vitamin A metabolism. Lipid metabolism affects the availability of retinol, a precursor of RA, as retinol is primarily stored in the form of retinyl esters [8–11]. Vitamin A metabolism is further linked to central metabolism (e.g., citric acid cycle, glycolysis) through energy molecules such as NADPH and NADH. In addition, all these pathways are essential during differentiation of other cell types [12]. Lipid metabolism was shown to determine self-renewal or differentiation of hematopoietic stem cells [13]. Glycolysis is the primary energy source in many stem cell populations. As stem cells differentiate, the activity of anaerobic glycolysis decreases (i.e., fermentation of pyruvate to lactate) while the citric acid (TCA) cycle takes over the energy production [12]. However, it is unknown how these metabolic pathways function during spermatogonial differentiation.

These knowledge gaps are likely due to the enormous effort required to perform germ cell-specific gene knockouts (KOs) and measure metabolic activity within the germ cell. Individual gene KOs in the vitamin A pathway often yield no phenotype [8, 9, 14]. Double or triple KOs are difficult to obtain but would be needed to elucidate the role of vitamin A metabolism in generating the RA pulse. Investigation of metabolic pathways would require direct measurement of enzyme activities and metabolite levels.

Because germ cell metabolism is highly complex, a thorough understanding cannot be achieved by only studying individual genes in animal models. A more profound understanding requires computational models to integrate genome-scale and individual studies of enzymes, metabolites, and reactions into networks of interacting components. A metabolic network model can reveal key players by performing *in silico* perturbations of enzymes and reactions and predict system-wide outcomes on a scale that would be impossible by *in vivo* approaches [15–18]. We developed a mouse germ cell-specific metabolic network. A manually curated vitamin A pathway was integrated into the network to ensure accurate representation. Flux balance analysis (FBA) was performed on the network constrained with germ cell-specific gene expression [19]. With this *in silico* platform, we predict enzymes and reactions that are imperative for controlling RA levels within the germ cell. Further, we identify active pathways and energy producing pathways in the germ cell. This study provides a comprehensive understanding of how global metabolism regulates RA availability and germ cell differentiation.

## Results

### Construction of a germ cell-specific metabolic network

#### Vitamin A metabolic pathway

**Fig 1:**
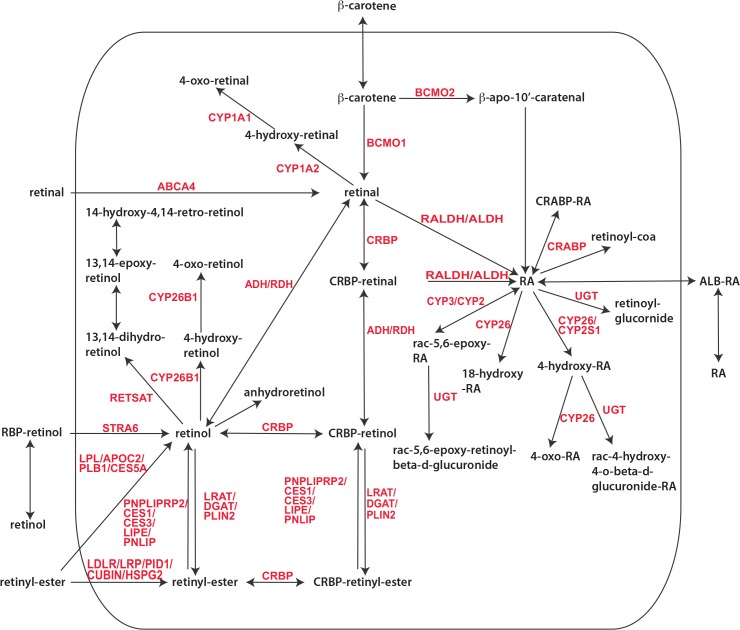
Vitamin A metabolic pathway. Reactions involved in retinoid metabolism are depicted. Exchange/sink reactions and promiscuous metabolites (e.g., energy molecules, O_2_, H_2_O) are omitted from the diagram. The rounded rectangle denotes germ cell compartment.

We compiled a list of metabolites and genes known to be involved in vitamin A metabolism (S1 Table). Reactions containing these metabolites or genes were extracted from human and mouse metabolic databases including Recon1, Recon2, EHMN, Reactome, and BioCyc [20–24]. Human reactions were considered to exist in the mouse if the catalyzing human enzymes had mouse orthologs [25]. We manually curated the extracted reactions by standardizing metabolite and gene names, eliminating reactions specifically in the vision cycle, and removing redundant reactions. Isomers (i.e., 9-cis, 11-cis, and 13-cis) of retinol, retinal, and RA were excluded, as our goal was to discover enzymes and reactions in germ cells that may contribute to the pulse of all-trans RA detected experimentally [5]. Different forms of retinyl-esters (i.e., retinyl-palmitate, fatty acid retinols, and retinyl-esters) were condensed into one set of metabolites. Reactions were assigned to a sub-cellular compartment: cytoplasm, nucleus, or extracellular region. The resulting vitamin A pathway comprises 45 reactions (13 reversible, 26 irreversible, and 6 exchange/sink reactions), 79 genes, and 44 metabolites (Fig 1).

#### Germ cell-specific metabolic network

We constructed a genome-scale mouse metabolic network from human Recon2 [21] by following a similar procedure described previously [26]. Specifically, human genes were converted into mouse orthologs; reactions with mouse orthologs or previously without human gene association were included in the mouse network. The resulting network was tested iteratively until it passed a set of 260 validation tests, which ensured necessary metabolites and biomass could be produced.

A germ cell-specific network was constructed by extracting pathways potentially active in germ cells from the mouse genome-scale network. We utilized time-course germ cell-specific gene expression during the first wave of spermatogenesis [19]. Specifically, mice were treated with the compound WIN 18,446 for nine days starting from 1-day post-partum (dpp). WIN 18,446 inhibits the conversion of retinal to RA by targeting aldehyde dehydrogenases [27]. At 9 dpp, mice were given an injection of RA to initiate the synchronized first wave of spermatogenesis. Testes were collected at 0, 4, 12 hours, 1, 2, 4, 6, 8 days after the RA injection for expression profiling. This protocol ensures the enrichment of specific germ cell types during the first wave. Undifferentiated spermatogonia were initially enriched as a result of WIN 18,446 treatment whereas A differentiating spermatogonia were abundant at 4 hours through 2 days post-RA injection. Intermediate spermatogonia were enriched at 4 days. Preleptotene and leptotene spermatocytes accumulated by 6 and 8 days post-RA injection, respectively. In addition, A differentiating spermatogonia from the second wave of spermatogenesis were also enriched at 6 and 8 days (S1 Fig) [19]. Expression data were obtained by measuring ribosome-associated mRNAs in the germ cell. By capturing the level of mRNAs potentially being translated into proteins, this method approximates enzyme activity better than monitoring total mRNAs [28]. In addition, this method captures germ cell-specific gene expression. Note that the WIN 18,446/RA protocol used to profile gene expression was similarly implemented to detect the RA pulse during spermatogonial differentiation [5]. Thus, it is appropriate to use this expression data to predict enzymes and reactions in germ cells that may contribute to the observed RA pulse.

To extract potentially active pathways in the germ cell, we first classified genes in the mouse genome-scale network as “highly expressed” or “lowly expressed” using the expression data described above. A gene is considered “highly expressed” in the germ cell if its expression value is above the median value of all genes across all time points. Conversely, a gene is considered “lowly expressed” if its expression value is below the median value. A metabolic pathway is considered active if it is significantly enriched for reactions that are associated with “highly expressed” genes (hypergeometric P-value < 0.05). Reactions without gene associations were excluded prior to the P-value calculation.

**Fig 2:**
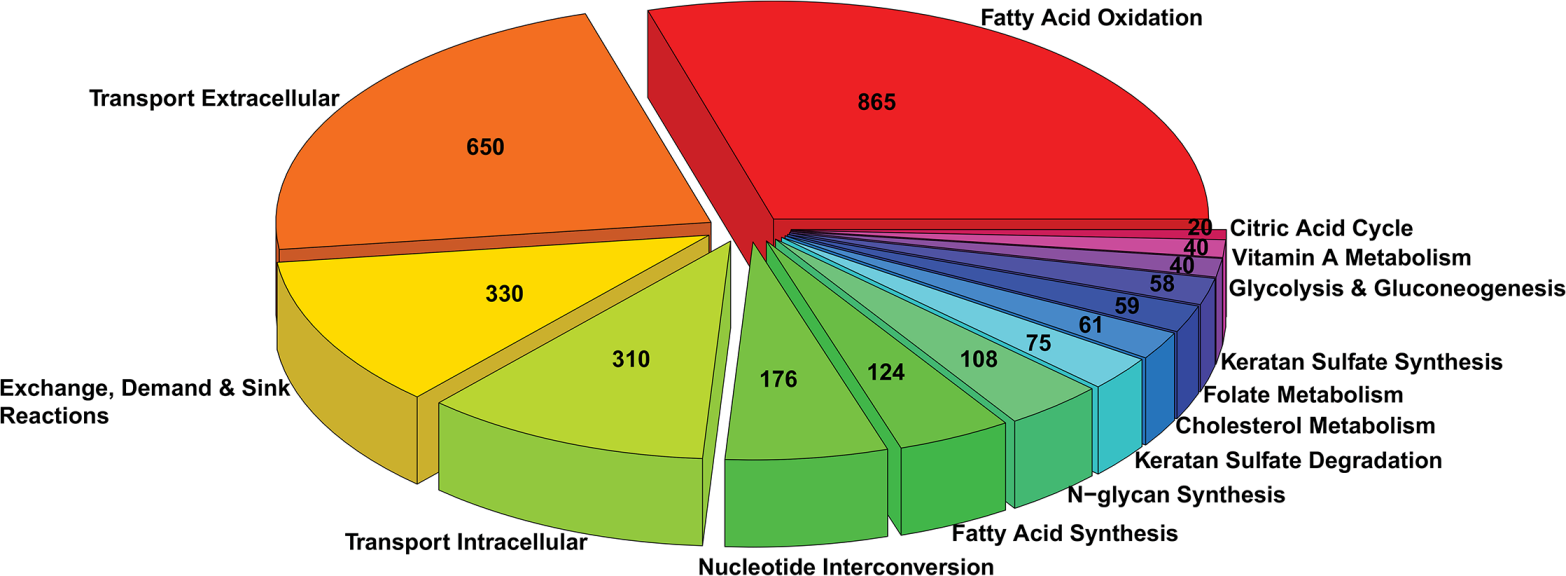
A mouse germ cell-specific metabolic network. Reactions in the network are grouped into pathways. The number of reactions in each pathway is labeled.

Utilizing this procedure, 10 out of 92 metabolic pathways (excluding transport and exchange reactions) were identified as active in the germ cell for at least one time point: cholesterol metabolism, TCA, fatty acid oxidation, fatty acid synthesis, folate metabolism, glycolysis and gluconeogenesis, keratan sulfate degradation, keratan sulfate synthesis, N-glycan synthesis, and nucleotide interconversion. Vitamin A metabolism was added as the 11^th^ pathway because of its important role in spermatogonial differentiation [2–4]. Then extracellular transport reactions were incorporated if they contain at least one metabolite in the active pathways. Intracellular transport and exchange reactions were incorporated if all metabolites in the reactions are in the active pathways. These pathways and reactions were combined to generate a germ cell-specific compartmentalized mouse network. The network is capable of producing biomass without any modifications. We cast the network in the SBML format to facilitate model sharing (S1 File). In addition, we provide Cytoscape session files [29] to visualize the network (S2 File). A total of 2,916 reactions (1,597 reversible and 1,319 irreversible) in eight sub-cellular compartments (cytoplasm, nucleus, mitochondria, endoplasmic reticulum, Golgi apparatus, peroxisome, lysosome, and extracellular region) are captured in the network, with 636 genes and 2,072 metabolites (Fig 2).

### Dynamic flux of the vitamin A metabolic pathway

Using the germ cell-specific network, we simulated network flux (i.e., reaction rate) at eight time points during the first wave of spermatogenesis. As not all enzymes are expressed at a given time, we used the germ cell-specific gene expression [19] to define the maximum allowable flux for reactions (i.e., reaction constraints). This was performed by mapping the expression of genes to their catalyzed reactions in the network. Therefore, if an enzyme is lowly expressed, its associated reaction would have low activity. Conversely, a highly expressed enzyme would indicate that the associated reaction has the potential to be active. Then at each time point, reaction fluxes were computed by Markov chain Monte Carlo sampling implemented with the COBRA Toolbox [30]. The fluxes values are influenced by both network structure and expression-defined reaction constraints. Using the network model, we directly investigated intracellular RA availability.

**Fig 3:**
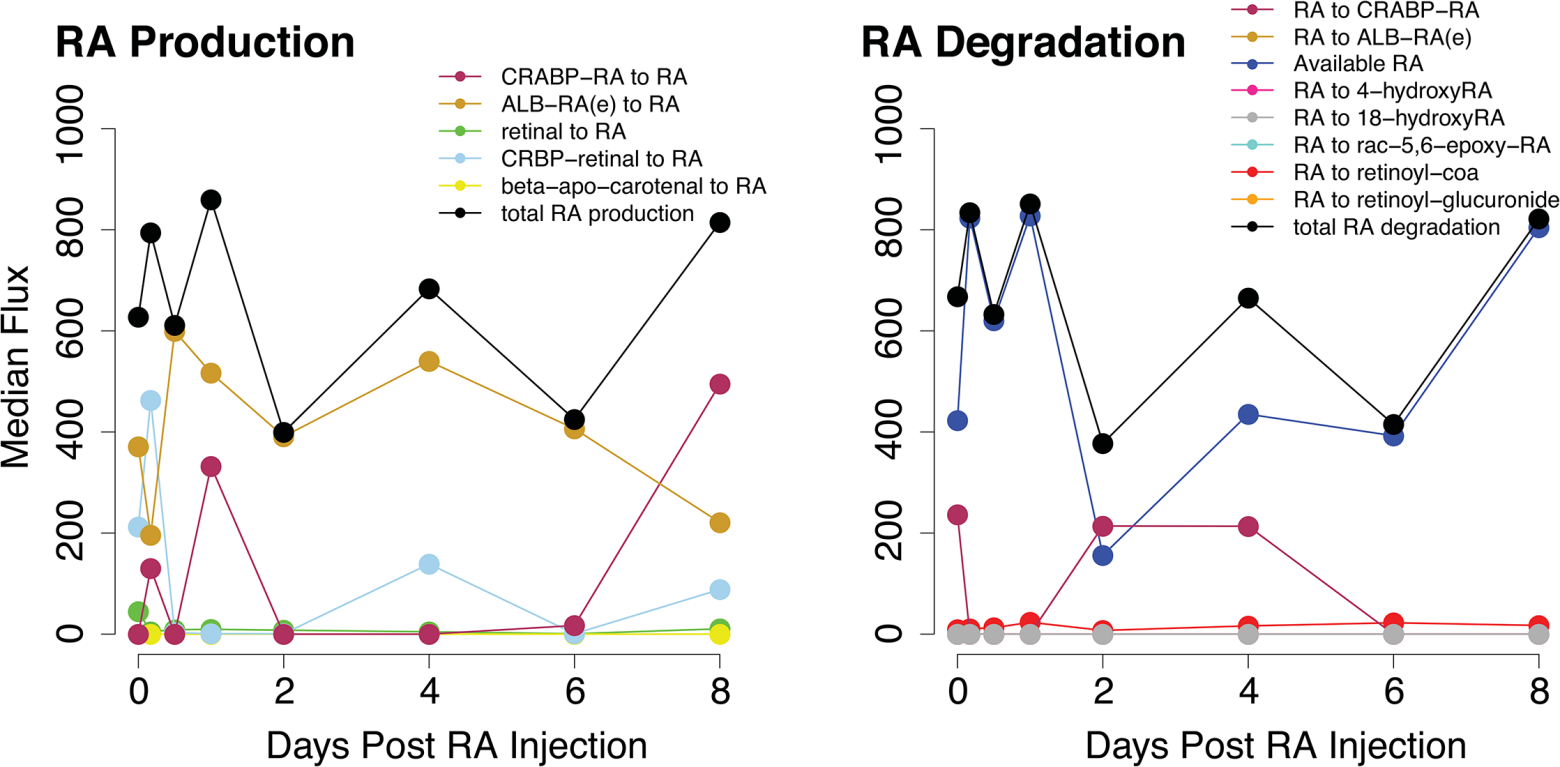
Flux of reactions that directly produce or degrade RA. Median flux values from the model are plotted across eight days after initiation of spermatogenesis via an RA injection.

Our result shows that total RA production exhibits two peaks: an initial peak from 4 to 24 hours and a second peak on day eight post-RA injection (Fig 3). The initial RA peak is derived primarily from import from the extracellular region, in addition to internal metabolism from CRBP-retinal and RA unbinding from CRABP. Because the first wave of spermatogenesis was initiated by an injection of RA in the mice [19], it is reasonable to observe from the model that extracellular import is the major RA source. The same three reactions are also responsible for the peak on day eight. However, RA release from the binding protein CRABP becomes the dominant source, suggesting the binding reaction is essential for regulating RA levels within the germ cell.

RA degradation also peaks on day one and eight post-RA injection (Fig 3). Our results indicate that cytochrome P450 enzymes contribute minimally to RA degradation in the germ cell throughout the first eight days of spermatogenesis. This observation is supported by experimental evidence that *Cyp26* was detected in other cell types but not germ cells in the testis [31, 32]. RA binding to CRABP contributes more to the reduction of RA, specifically between 2 and 4 days post-RA injection. We proceeded to evaluate freely available RA in the germ cell with a sink reaction. Sink reactions are unbalanced network reactions that allow the accumulation of metabolites. The flux of this sink reaction measures RA being produced but not degraded. Available RA in the cytoplasm serves as an indicator of spermatogonial differentiation as RA initiates differentiation by being transported into the nucleus and binding to nuclear receptors [8, 9, 10, 33]. We observe high levels of available RA on day 1 and 8 post-RA injection, which is consistent with experimental observations that showed a pulse of RA at those time points [5].

We further examined reactions that control the levels of retinol and retinal, two RA precursors (S2 Fig). We find that cellular uptake of retinol via *Stra6* is minimal throughout the first eight days of spermatogenesis. This is consistent with experimental evidence that *Stra6* is not expressed in germ cells and global *Stra6* KO shows no spermatogenic defect [34, 35]. In addition, our model shows low activities of retinol esterification catalyzed by *Lrat*, suggesting that retinol storage does not occur in germ cells. This is supported by experimental data that *Lrat* is not expressed in germ cells [31, 32]. Further, we discover that retinol and retinal levels are directly regulated by the reversible reaction between them and binding reactions with CRBP. Our results show that a group of reactions form a loop to regulate retinol and retinal levels. At 12 hours post-RA injection, this loop works by unbinding retinal from CRBP and converting it to retinol. Next, retinol is bound to CRBP and converted to CRBP-retinal. The loop reverses the direction on day 1, and then flips back on day 4 post-RA injection. This loop maintains a supply of retinal that can be metabolized for RA production.

### Model prediction of RA enzymes and reactions required for spermatogenesis

#### Identification of criteria to predict spermatogenic defects

Using the tractable computational model, we can perturb enzymes and reactions individually or collectively. These virtual experiments allow us to overcome genetic redundancy to predict germ cell-specific enzymes and reactions that are most important for controlling RA levels. Two statistical tests were used to assess the difference between wild type (WT) (previous simulations in the subsection of “Dynamic flux of the vitamin A metabolic pathway”) and perturbation simulations. The Spearman correlation coefficient measures differences in global network flux values between WT and perturbation simulations. The Kolmogorov-Smirnov (K-S) statistic measures differences in available RA within the germ cell between WT and perturbation simulations. Perturbations are considered to cause spermatogenic defects if both network flux and available RA are different from those of WT simulation for at least one time point.

**Fig 4:**
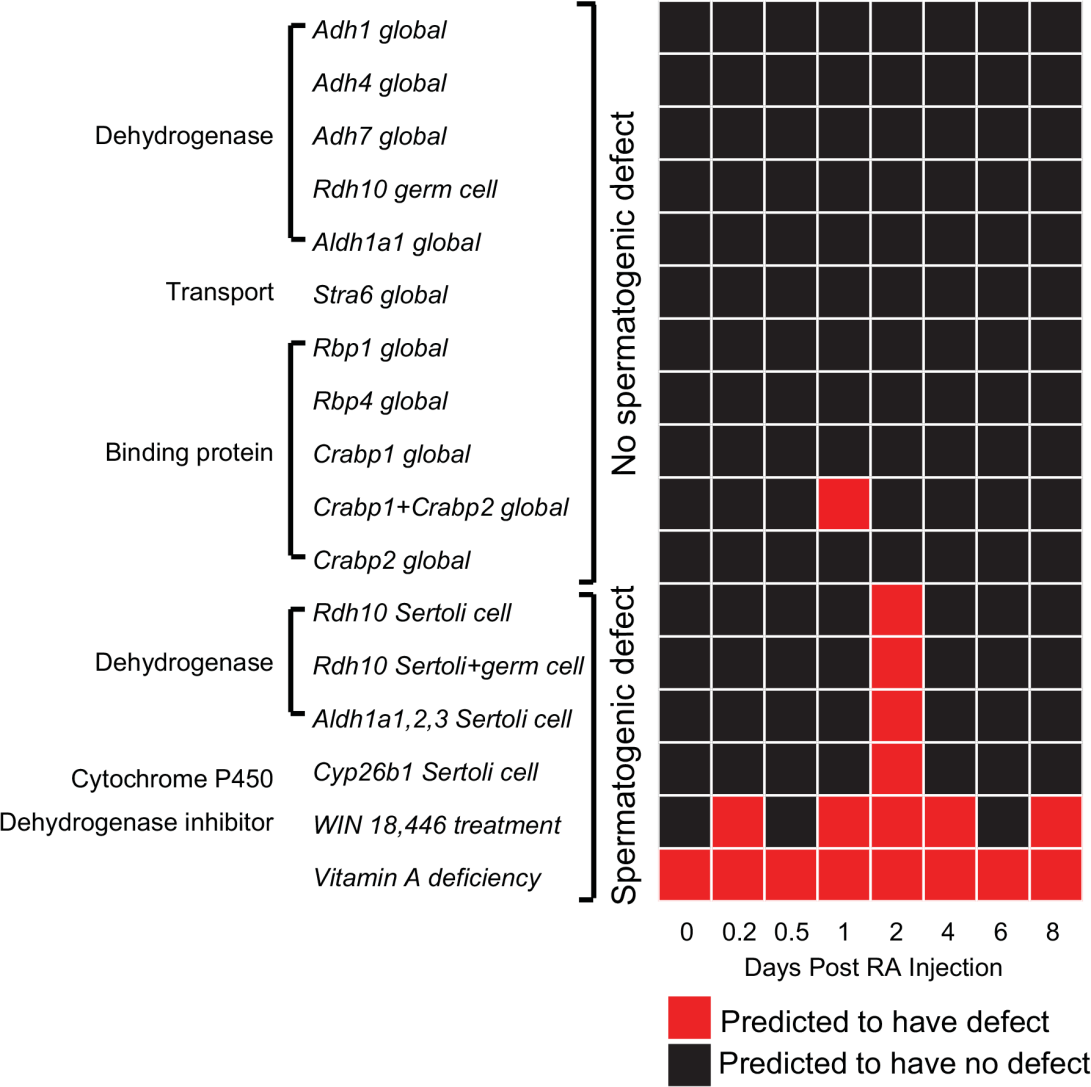
Identification of criteria to predict spermatogenic defects. A total of 17 experimental perturbations on male fertility are listed, including global KOs, cell-specific KOs, and compound treatments. Among these perturbations, six cause spermatogenic defects and 11 produce no defects. Using a Spearman coefficient less than 0.95 and a K-S statistic greater than 0.9 for at least one time point to predict defects, our model results match 16 out of 17 experimental perturbations.

To identify thresholds of the two statistical tests used to predict spermatogenic defects, we conducted *in silico* enzyme KOs and treatments for which the effect on spermatogenesis has been experimentally demonstrated. We collected a total of 17 experimental perturbations on retinoid metabolism, including global KOs, germ cell KOs, Sertoli cell KOs, and compound treatments [3, 6, 7, 35–43]. Among these experiments, six cause spermatogenic defects and 11 produce no defects (Fig 4). We simulated each of the 17 perturbations, taking into the account the origin of perturbation (e.g., global KO, cell-specific KO). All possible threshold combinations of the two statistical tests were evaluated. Using a Spearman coefficient less than 0.95 and a K-S statistic greater than 0.9 to define defects, our model achieved the best performance, replicating 16 out of 17 experimental results (Fig 4). All of the KOs and treatments causing spermatogenic defects were predicted correctly. Of the 11 no defect KOs, all but one (*Crabp1*+*Crabp2* global KO) were predicted correctly.

#### Prediction of new genes and reactions required for spermatogenesis

**Fig 5:**
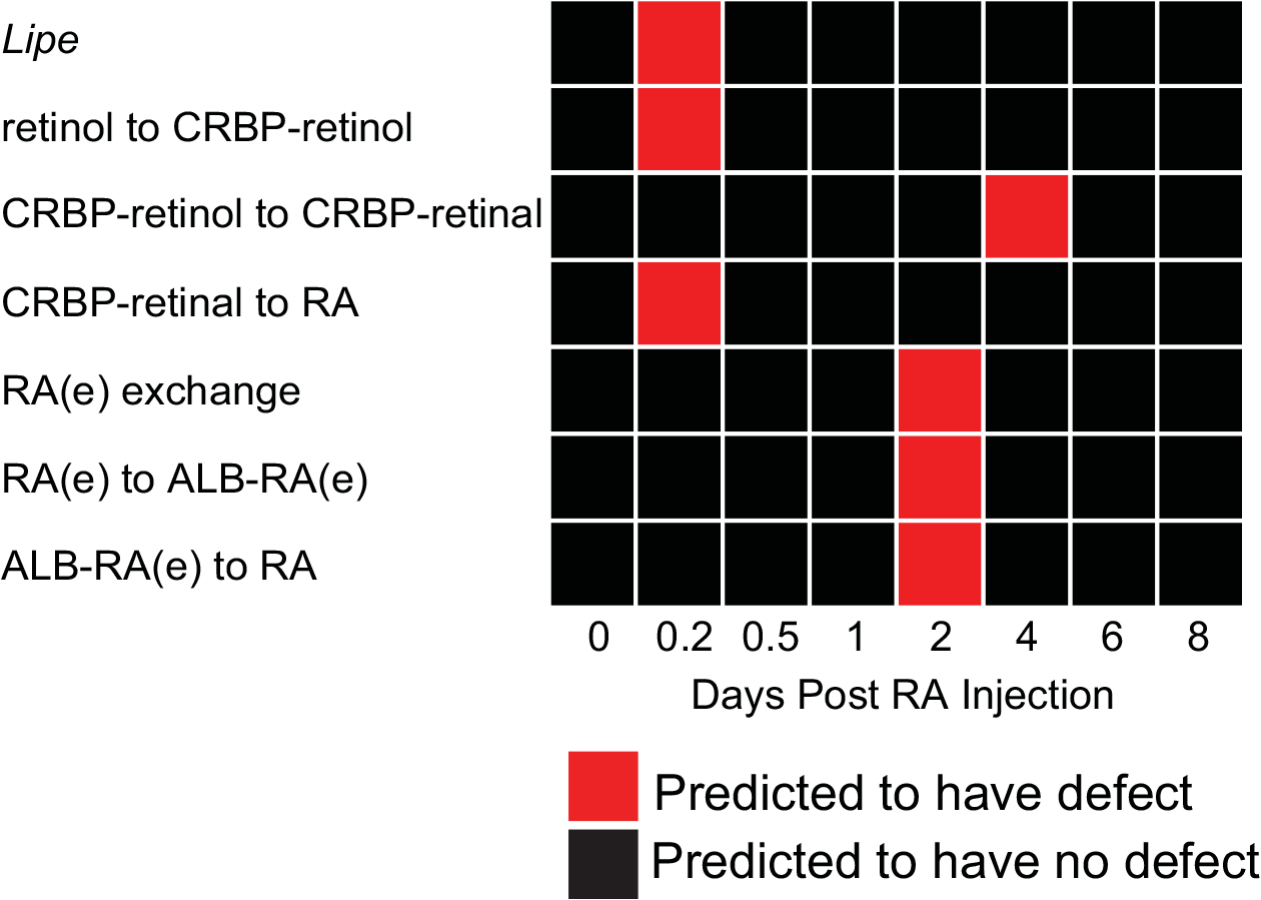
Genes and reactions predicted to be required for spermatogenesis. One gene and six reactions are predicted to cause spermatogenic defects, defined by having a Spearman coefficient less than 0.95 and a K-S statistic greater than 0.9 for at least one time point.

We simulated single gene KOs for all the genes in the vitamin A pathway that were not used to generate prediction thresholds. Our model predicts that deletion of only one gene, *Lipe*, would result in a spermatogenic defect at hour 4 post-RA injection (Fig 5). LIPE catalyzes two reactions in the model: conversion from retinyl-ester to retinol and conversion from CRBP-retinyl-ester to CRBP-retinol. *Lipe* KO causes minor flux changes in these two reactions. However, major increases in retinol being imported into the cell and metabolized into RA are observed. The end result is an increase in available RA within the germ cell (S3 Fig). We speculate that this increase is due to a compensation response from the cell to deal with the deletion of *Lipe*.

Similarly, we performed single reaction KOs for all reactions in the vitamin A pathway, and found six are predicted to be required for spermatogenesis using the thresholds determined previously (Fig 5). Two of these six reaction KOs cause an increase in available RA. When the retinol binding reaction is deleted, the network flux is altered at hour 4 post-RA injection. More retinol is transported into the cell and converted into retinal and RA (S4 Fig). This again causes an increase in available RA, indicating that the retinol-binding reaction is important for the homeostasis of the pathway. When the reaction of CRBP-retinol to CRBP-retinal is deleted, a spermatogenic defect is predicted on day 4. The alternative reaction from retinol to retinal is activated, thereby generating more available RA than in the WT (S5 Fig). This is a compensation response likely due to the disruption in the pathway homeostasis.

In contrast to the above two reaction KOs, the remaining four reaction KOs are predicted to cause spermatogenic defects due to a decrease in available RA. When CRBP-retinal to RA is deleted, a spermatogenic defect is predicted at hour 4 post-RA injection. This reaction is the major source of RA at that time (Fig 3), thus the deletion causes the reduction of available RA. Although RA import into the cell increases to compensate for the loss, the majority binds CRABP, further reducing the available RA (S6 Fig). Three reaction KOs (RA(e) exchange, RA(e) to ALB-RA(e), ALB-RA(e) to RA) all prevent RA from being imported into the cell. Extracellular RA is the sole RA source on day 2 (Fig 3), therefore deletion of any of the three reactions reduces the available RA to zero. Although these reaction KOs cause changes in retinol and retinal conversions, they are not metabolized to produce RA (S7 Fig).

Our reaction KO predictions are consistent with important reactions indicated by the dynamic flux of the vitamin A pathway (Fig 3, S2 Fig). Predicted reactions are major sources or sinks of retinoids in the germ cell. Disruption in these reactions either increases or decreases available RA; both can result in spermatogenic defects as reported experimentally [3, 6, 7, 36, 43].

### Activities of other pathways in the germ cell-specific metabolic network

**Fig 6:**
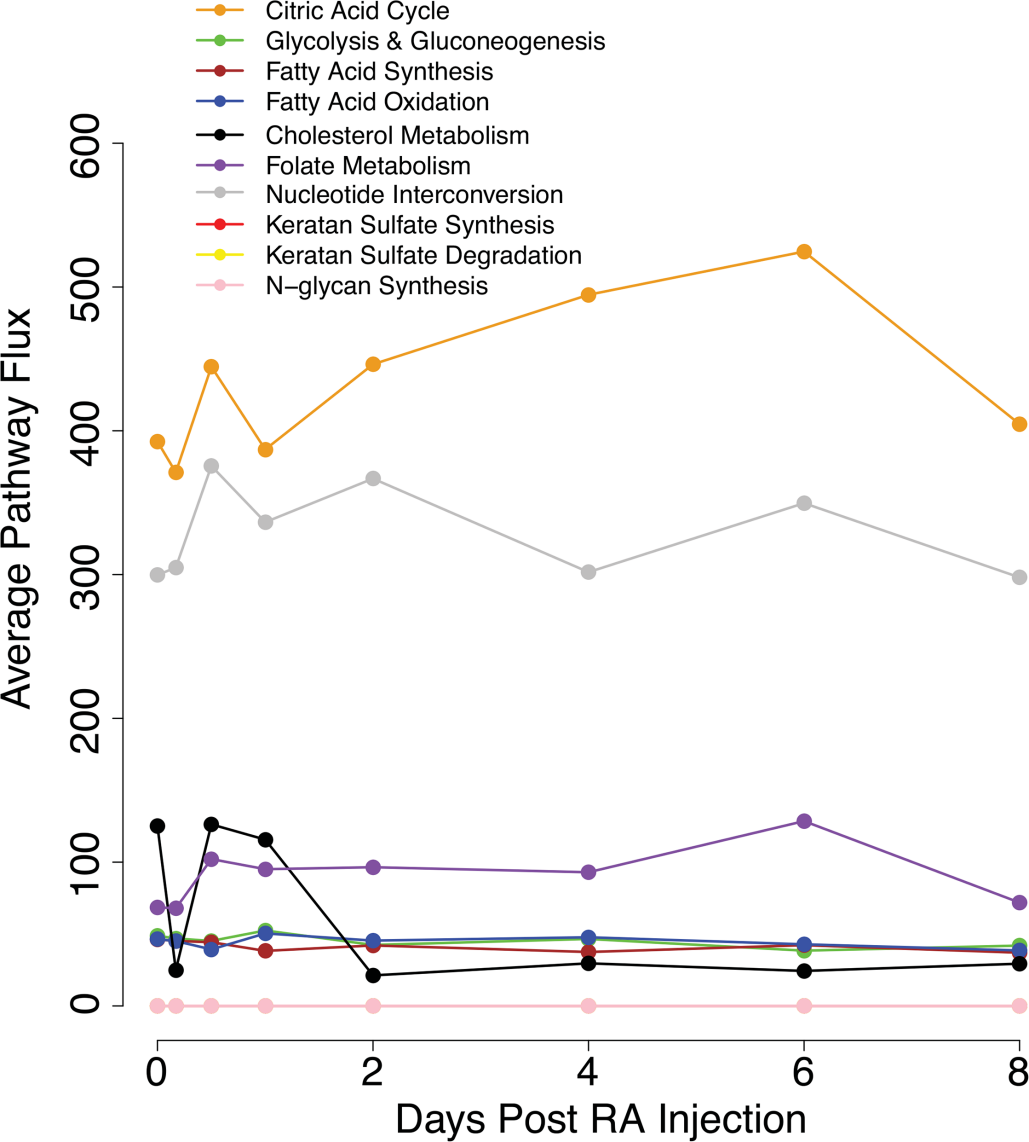
Activities of other pathways in the germ cell-specific metabolic network. The average pathway flux is calculated by summing the median flux values of each reaction and dividing by the number of reactions in a pathway at each time point.

Besides vitamin A metabolism, we examined the activity of the other ten pathways in the germ cell-specific metabolic network. Activity was quantified by averaging the median flux of reactions included in each pathway (Fig 6). The TCA cycle is the most active pathway, steadily increasing in activity until day 6 post-RA injection and then decreasing. Nucleotide interconversion is the second most active pathway, consisting of conversion reactions of mono-, di-, and tri-phosphate nucleotides. Because undifferentiated and differentiating spermatogonia are actively growing and mitotically dividing, they require nucleotides for incorporation into DNA. Folate metabolism, important for DNA replication and epigenetic modifications such as DNA methylation [44], has stable activity throughout the first eight days of spermatogenesis. The activity of cholesterol metabolism peaks within a day and then declines. Glycolysis and gluconeogenesis, fatty acid synthesis and degradation all have low metabolic activity during the first wave in germ cells. Three pathways, keratan sulfate synthesis, keratan sulfate degradation, and N-glycan synthesis, are completely inactive.

**Fig 7:**
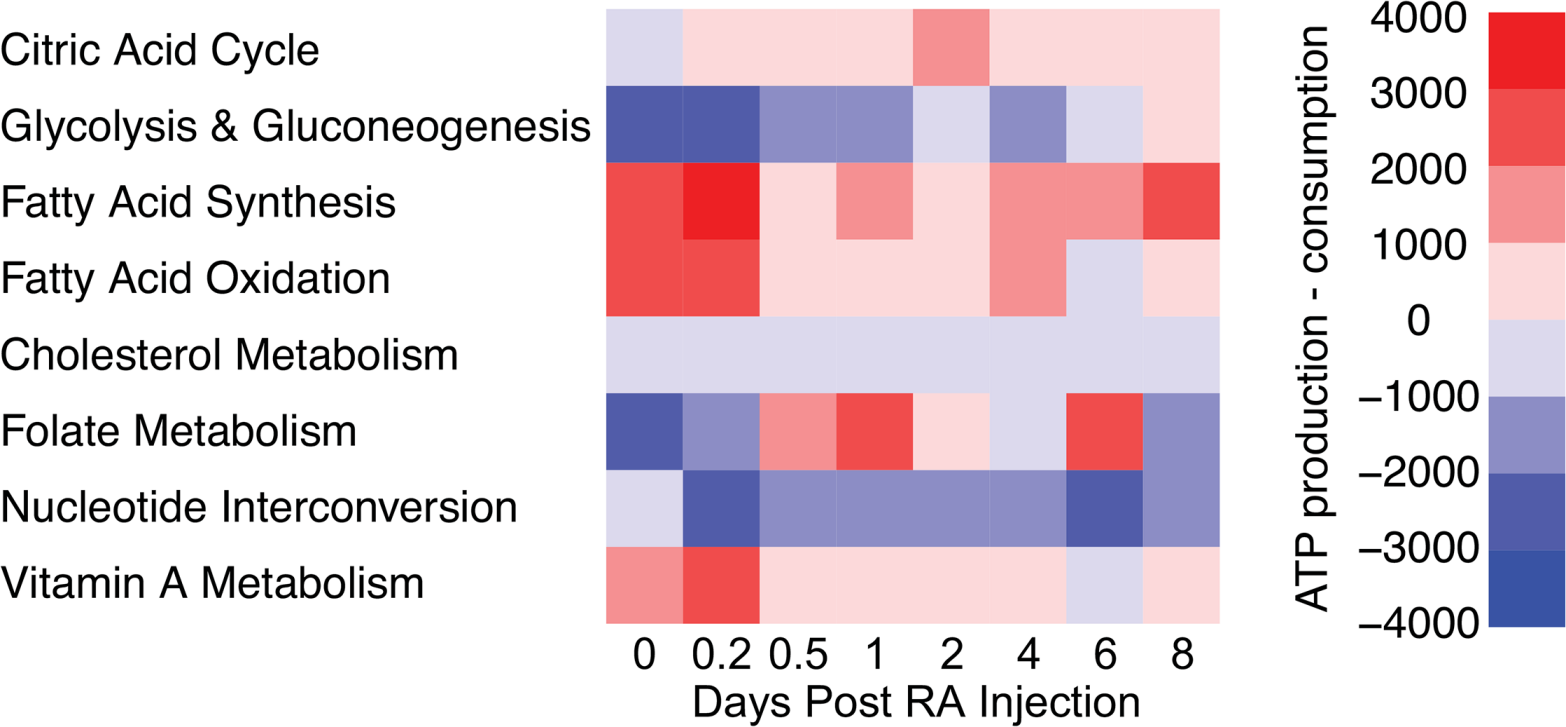
The contribution of individual pathways to energy production and consumption. All energy molecules are converted to the ATP unit: 1NADH = 3ATP, 1NADPH = 4ATP, 1FADH2 = 2ATP, 1GTP = 1ATP, 1CTP = 1ATP, 1TTP = 1ATP. Net ATP production is calculated for each pathway at each time point by subtracting ATP consumption from production. A positive value means a pathway generates energy while a negative value indicates the pathway consumes energy.

Because energy is vital for successful differentiation of cells, we further examined which metabolic pathways are producing and consuming energy (Fig 7). The three inactive pathways were excluded from this analysis. Our results show that fatty acid synthesis is the primary source of energy production in germ cells across the first wave of spermatogenesis. Highest energy yields occur at hour 4 and day 8 after initiation of spermatogenesis via an RA injection, corresponding to the timing of spermatogonial differentiation [45]. Fatty acid oxidation is the second highest energy-producing pathway, followed by vitamin A pathway and TCA cycle. Energy consuming pathways mainly include nucleotide interconversion, glycolysis and gluconeogenesis, and cholesterol metabolism.

### Metabolites important for germ cell differentiation

We investigated two metabolites known to be important for stem cell differentiation: pyruvate and lactate [12]. Pyruvate is mainly produced from glycolysis in stem cells and converted to lactate for anaerobic respiration. This prevents pyruvate from entering the TCA cycle, which induces differentiation [12]. As stem cells differentiate, pyruvate is shunted into the TCA cycle, resulting in decreased lactate production. Although our model does not capture stem cell differentiation, it describes a differentiation process of germ cells. We examined whether the metabolic trend of spermatogonial differentiation could be similar to that of stem cells.

**Fig 8:**
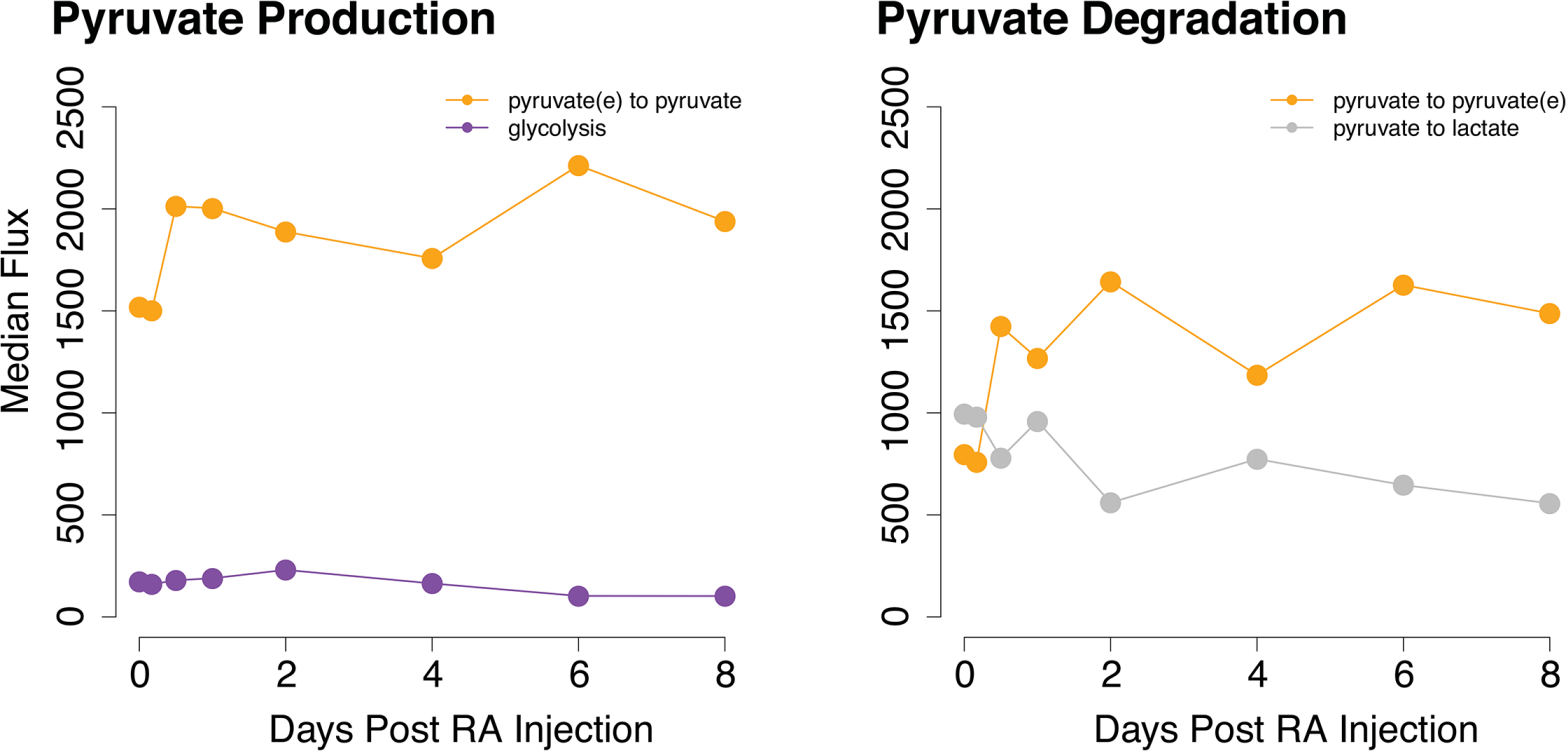
Flux of reactions that directly produce or degrade pyruvate. Median flux values from the model are plotted across eight days after initiation of spermatogenesis via an RA injection.

We find that pyruvate is primarily produced and consumed via transport in and out of the germ cell. Pyruvate production through glycolysis is extremely low (Fig 8). This is consistent with our earlier result of minimal glycolysis activity throughout the first eight days of spermatogenesis (Fig 6). Because it is known that glycolysis is highly active in Sertoli cells [46], and our results show that germ cell pyruvate primarily comes from the extracellular region, we speculate that pyruvate is mainly produced by Sertoli cells and transported into germ cells. Pyruvate is then converted into lactate. We observe a decrease in lactate production (Fig 8) and an increase in TCA activity during the first wave (Fig 6). This indicates that TCA activity and lactate production during spermatogonial differentiation follow the same trend as stem cell differentiation. The low glycolysis activity throughout the first wave, however, is unique to the germ cell.

## Discussion

RA is essential for germ cell differentiation [2, 3]. RA acts in a pulse manner, with concentration peaking every 8.6 days in the mouse thereby initiating spermatogonial differentiation [5]. We constructed a mouse germ cell-specific metabolic network. Using flux balance analysis with germ cell-specific gene expression, we reproduced the pulse of RA during the synchronized first wave of spermatogenesis (Fig 3). Our results show that multiple reactions contribute to the RA pulse in the germ cell, including import from extracellular region, metabolism from retinal, and RA unbinding from CRABP (Fig 3). Because RA was injected into mice to initiate the first wave [19], it is reasonable to observe that extracellular transport is the primary source of RA at early time points. Experimental evidence shows that Sertoli cells are primarily responsible for producing RA during the first spermatogenic wave [6, 7], and likely the reason we still observe extracellular transport as a major RA source at later time points. Metabolism of retinal and unbinding from CRABP are also important RA sources, suggesting that internal RA production contributes to the total RA levels in the germ cell. Further, we observe that RA is minimally degraded by reactions within the germ cell indicating that other cell types may be responsible for RA degradation.

Using the FBA model, we predict genes and reactions that are critical for regulating RA availability (Fig 5). Overall, few gene and reaction KOs in the germ cell were predicted to cause spermatogenic defects, likely because of genetic redundancy in the vitamin A pathway or activity of neighboring somatic cells [6, 7]. *Lipe* was the only gene predicted to have an important role in regulating RA levels. Previous experimental studies show that global KO of *Lipe* in mice causes major defects in round spermatids and a decreased sperm count leading to infertility [47–49]. Spermatid abnormalities were evident as early as five weeks after birth [49]. However, the mechanism of infertility was not thoroughly investigated. One study speculated that a metabolite downstream from *Lipe* reaction might be essential for membrane integrity in germ cells [49]. Here we show that, *in silico*, *Lipe* KO results in an increase in available RA, altering the regulation of spermatogonial differentiation in the first wave. Although phenotypic defects were not observed at this stage in *Lipe* KO mice, mis-regulation of vitamin A metabolism may affect downstream spermatid development and may only be phenotypically evident until later stages of spermatogenesis.

In addition, six reaction KOs are predicted to cause spermatogenic defects (Fig 5). Three of these reactions transport RA into the germ cell, further suggesting that somatic cells are responsible for generating the RA pulse. While investigation into the role of Sertoli cells has started [6, 7], other cells such as Leydig and peritubular myoid cells may also be important. The three remaining reaction KOs are involved in vitamin A metabolism in germ cells. Specifically, disruption in retinol to CRBP-retinol and in CRBP-retinol to CRBP-retinal increases RA levels while disruption of CRBP-retinal to RA decreases RA levels. Together, these results from our model suggest that although somatic cells are the major regulator of RA pulse, vitamin A metabolism in the germ cell also contributes to RA levels, and consequentially, spermatogonial differentiation.

Other pathways in our model have been examined to understand their roles in spermatogonial differentiation. The TCA cycle is the most metabolically active pathway, increasing in activity during the first wave of spermatogenesis (Fig 6). This occurs concurrently with decreased production of lactate (Fig 8), consistent with the metabolic trend observed in stem cell differentiation [12]. Pyruvate, the end product of glycolysis and the precursor to TCA and lactate, is primarily obtained from the extracellular region (Fig 8). This is unsurprising as glycolysis activity in the germ cell is low throughout the first wave (Fig 6). Experimental evidence indicates that Sertoli cells have high rates of glycolysis and provide pyruvate and other essential metabolites to spermatocytes [46]. Therefore, our results suggest that Sertoli cells are likely the source of glycolysis intermediates for germ cells. In addition, our model predicts that fatty acid metabolism is the primary producer of energy throughout the first wave of spermatogenesis (Fig 7). Energy production peaks concurrently with the timing of the RA pulse and spermatogonial differentiation. This indicates that fatty acid metabolism may indirectly affect the RA pulse by controlling the availability of co-factors and energy molecules required for reactions. This interesting finding from our model will require experimental investigation to further elucidate connections between fatty acid metabolism and vitamin A metabolic pathway.

In summary, our FBA model constitutes a powerful tool for manipulating genes and reactions to determine their contribution to vitamin A metabolism in the germ cell. These *in silico* perturbations prioritize cell-specific enzymes and reactions based on quantitative flux metrics, thus providing directions for future functional studies. Further, our FBA model traces metabolic activities of other pathways, allowing for the identification of pathways important for energy production and therefore spermatogonial differentiation. Future improvements of the computational approach will include integrating genome-scale metabolic networks of multiple cell types (e.g., germ, Sertoli, and Leydig cells) in the testis to elucidate how enzymes/reactions in other cells affect RA availability in germ cells.

## Materials and Methods

### Flux balance analysis

FBA calculates the flow of metabolites through a network under the assumption that each metabolite has equal production and consumption. All reactions in the network are represented in a stoichiometric matrix, *S*_*m* × *r*_, where *m* is the number of metabolites and *n* is the number of reactions. Matrix entries are metabolite coefficients in each reaction. Constraints are placed upon each reaction: *V*_min_ ≤ *V* ≤ *V*_max_, defining the range of allowable flux values. Steady state analysis is carried out by setting mass balance equations equal to zero: *S*_*m* × *r*_ *V* = 0.

### FBA constraints

Maximum and minimum constraints were placed on each reaction in the network. Germ cell-specific gene expression was used to define the maximum flux. Briefly, 1 dpp mice were fed WIN 18,446 for 9 consecutive days. At 9 dpp, mice were injected with RA to initiate spermatogenesis. Testes were collected at 0, 4, 12 hours, 1, 2, 4, 6, 8 days post-RA injection for expression profiling. Ribosome associated mRNAs within germ cells were pulled down to evaluate global gene expression (GSE54408) [19]. Microarray data were normalized using the Robust Multi-array Average (RMA) method. The probe IDs were translated into Ensembl gene IDs based on the Mouse ST Gene 1.0 transcript annotation version 33.2. In the case of multiple probe IDs for one gene, the average expression value was used. Finally, signals from duplicate samples were averaged to yield the final gene expression value. Expression values for the 636 genes in the network were extracted. If a reaction is catalyzed by multiple isozymes, the maximum constraint was assigned with the value of the highest expressed isozyme. If a reaction is catalyzed by enzyme complexes, the maximum constraint was assigned with the value of the lowest expressed complex subunit. Once maximum constraints are determined for reactions associated with enzymes, they were scaled to have an average of 1,000 over the eight time points. For reactions without enzyme association or without expression data, the average of maximum constraints in the network was used.

Minimum constraints were determined based on reaction reversibility; irreversible reactions were given a value of zero while reversible reactions were assigned the negative value of the maximum constraint. For intracellular binding reactions of retinyl-ester, retinol, retinal, and RA, the minimum constraint was the maximum constraint at the time point subtracted by the highest maximum constraint across the eight time points.

### Sampling analysis of the network flux

Monte Carlo Markov chain sampling was used to acquire the distribution for all possible flux states. This was performed using a hit-and-run algorithm—gpSampler in the Cobra Toolbox [30]. At each time point with expression-based constraints and network topology, the solution space was sampled with 6,000 points for 17 minutes. This results in 6,000 feasible solutions for all reactions acquired from the uniformly sampled points. From this distribution, the median flux value was obtained for each reaction.

### Enzyme and reaction deletion analysis

To simulate germ cell-specific enzyme deletion, the expression of the enzyme was set to zero, and the maximum constraints were re-calculated for reactions catalyzed by this enzyme. To simulate germ cell-specific reaction deletion, the maximum and minimum constraints were both set to zero. Enzyme deletion in the Sertoli cell was simulated by altering the extracellular availability of metabolites through exchange reactions. Specifically, Sertoli KOs of *Rdh10* and *Aldh1a1*,*2*,*3* were mimicked by setting the minimum constraint of RA exchange reaction to zero, thus eliminating RA from the extracellular region. Conversely, Sertoli KO of *Cyp26b1* was mimicked by setting the maximum constraint of RA exchange reaction to -900, thus forcing RA to be present in the extracellular region. WIN 18,446 treatment was reproduced by setting maximum constraints to zero for all reactions catalyzed by aldehyde dehydrogenases, in addition to setting the minimum constraint of RA exchange reaction to zero. To replicate vitamin A deficiency, minimum constraints were set to zero for exchange reactions of any form of vitamin A (retinol, retinal, β-carotene, and RA). After the network was manipulated for each perturbation, candidate states were re-sampled and compared with normal candidate flux states by calculating the Spearman correlation coefficient and the K-S statistic.

## Supporting Information

S1 Fig Types of germ cells enriched at different time points post-RA injection. Mice were treated with WIN 18,446 for nine days starting from 1 dpp. At 9 dpp, mice were given an injection of RA to initiate spermatogonial differentiation.
(EPS)

S2 Fig Flux of reactions that directly produce or degrade retinol and retinal. Median flux values from the model are plotted across eight days after initiation of spermatogenesis via an RA injection.
(EPS)

S3 Fig Flux changes in the vitamin A pathway when *Lipe* is deleted *in silico* four hours after initiation of spermatogenesis via an RA injection. **A.** Difference in median flux values between WT and *Lipe* KO for reactions in the vitamin A pathway. Reactions with difference less than 50 are not shown. **B.** Sampling flux distribution for the reaction of available RA.
(EPS)

S4 Fig Flux changes in the vitamin A pathway when the reaction retinol to CRBP-retinol is deleted *in silico* four hours after initiation of spermatogenesis via an RA injection. **A.** Difference in median flux values between WT and this reaction KO for reactions in the vitamin A pathway. Reactions with difference less than 50 are not shown. **B.** Sampling flux distribution for the reaction of available RA.
(EPS)

S5 Fig Flux changes in the vitamin A pathway when the reaction CRBP-retinol to CRBP-retinal is deleted *in silico* four days after initiation of spermatogenesis via an RA injection. **A.** Difference in median flux values between WT and this reaction KO for reactions in the vitamin A pathway. Reactions with difference less than 50 are not shown. **B.** Sampling flux distribution for the reaction of available RA.
(EPS)

S6 Fig Flux changes in the vitamin A pathway when the reaction CRBP-retinal to RA is deleted *in silico* four hours after initiation of spermatogenesis via an RA injection. **A.** Difference in median flux values between WT and this reaction KO for reactions in the vitamin A pathway. Reactions with difference less than 50 are not shown. **B.** Sampling flux distribution for the reaction of available RA.
(EPS)

S7 Fig Flux changes in the vitamin A pathway when RA import into the cell is deleted *in silico* two days after initiation of spermatogenesis via an RA injection. Three single reaction deletions yielded the same result: RA(e) exchange, RA(e) to ALB-RA(e), and ALB-RA(e) to RA. **A.** Difference in median flux values between WT and single reaction KO for reactions in the vitamin A pathway. Reactions with difference less than 50 are not shown. **B.** Sampling flux distribution for the reaction of available RA.
(EPS)

S1 File The mouse germ cell-specific metabolic network in the SBML format. (XML)

S2 File Visualization of the mouse germ cell-specific metabolic network with Cytoscape. Cytoscape software can be downloaded from http://www.cytoscape.org/download.php. Visualization is provided for the full network as well as ten metabolic pathways: cholesterol metabolism, TCA, fatty acid oxidation, fatty acid synthesis, folate metabolism, glycolysis and gluconeogenesis, keratan sulfate degradation, keratan sulfate synthesis, N-glycan synthesis, and nucleotide interconversion. Pink diamonds represent reactions, gray circles depict metabolites, and green squares represent enzymes. Reversible reactions are linked to substrate or product metabolites with red edges whereas irreversible reactions are linked to metabolites with blue edges. Enzymes are linked to reactions with yellow edges.
(ZIP)

S1 Table Metabolites and genes known to be involved in vitamin A metabolism from the literature. (DOC)
