# Supplementary material for: Dissecting Germ Cell Metabolism through Network Modeling
Source: PLoS One. 2015 Sep 14;10(9):e0137607. doi: 10.1371/journal.pone.0137607 (PMC4721539; doi:10.1371/journal.pone.0137607)
Supplement: S1 Table — (DOC) [file pone.0137607.s010.doc]

**Table S1. Metabolites and genes known to be involved in vitamin A metabolism from the literature [1,2,3,4,5,6,7,8,9,10,11,12,13,14,15]**.

| **Metabolite** |
| --- |
| (2E,4E,6E,8E)-3,7-dimethyl-9-(2,6,6-trimethyl-1-cyclohexenyl)nona-2,4,6,8-tetraenyl hexadecanoate |
| (2E,4E,6E,8E)-3,7-dimethyl-9-(2,6,6-trimethylcyclohex-1-en-1-yl)nona-2,4,6,8-tetraen-1-yl acetate |
| (2E,4E,6E,8E)-3,7-dimethyl-9-(2,6,6-trimethylcyclohex-1-enyl)nona-2,4,6,8-tetraen-1-ol |
| (2E,4E,6E,8E)-3,7-dimethyl-9-(2,6,6-trimethylcyclohexen-1-yl)nona-2,4,6,8-tetraenal |
| (2E,4E,6E,8E)-3,7-dimethyl-9-(2,6,6-trimethylcyclohexen-1-yl)nona-2,4,6,8-tetraenoic acid |
| 4-oh-RA |
| 4-oxo-RA |
| acetylretinol |
| all-trans-retinal |
| all-trans-retinene |
| all-trans-retinol |
| all-trans-retinol acetate |
| all-trans-retinyl acetate |
| all-trans-Vitamin A acetate |
| all-trans-Vitamin A aldehyde |
| beta-carotene |
| retinal |
| retinaldehyde |
| retinoate |
| retinoic acid |
| retinol |
| retinol acetate |
| retinol palmitate |
| retinyl |
| retinyl-ester |
| retinyl acetate |
| retinyl palmitate |
| trans-retinol |
| Vitamin A |
| Vitamin A acetate |
| Vitamin A acid |
| Vitamin A palmitate |
| Vitamin A1 |
| Vitamin A1 acetate |
| Vitamin A aldehyde |
| **Gene** |
| *Abca* |
| *Adh* |
| *Alb* |
| *Aldh* |
| *Arat* |
| *Bcmo* |
| *Cel* |
| *Crabp* |
| *Cyp26* |
| *Dgat1* |
| *Hspg* |
| *Ldlr* |
| *Lipe* |
| *Lpl* |
| *Lrat* |
| *Lrp* |
| *Plin2* |
| *Plrp2* |
| *Pnlip* |
| *Raldh* |
| *Rbp* |
| *Rdh* |
| *Scarb* |
| *Stra6* |
| *Vldlr* |

**References**

1. Blaner WS, Obunike JC, Kurlandsky SB, al-Haideri M, Piantedosi R, et al. (1994) Lipoprotein lipase hydrolysis of retinyl ester. Possible implications for retinoid uptake by cells. J Biol Chem 269: 16559-16565.

2. Blomhoff R, Blomhoff HK (2006) Overview of Retinoid Metabolism and Function. J Neurobiol 66: 606-630.

3. Wei S, Lai K, Patel S, Piantedosi R, Shen H, et al. (1997) Retinyl ester hydrolysis and retinol efflux from BFC-1beta adipocytes. J Biol Chem 272: 14159-14165.

4. Kawaguchi R, Yu J, Honda J, Hu J, Whitelegge J, et al. (2007) A membrane receptor for retinol binding protein mediates cellular uptake of vitamin A. Science 315: 820-825.

5. During A, Dawson HD, Harrison EH (2005) Carotenoid Transport Is Decreased and Expression of the Lipid Transporters SR-BI, NPC1L1, and ABCA1 Is Downregulated in Caco-2 Cells Treated with Ezetimibe. J Nutr 135: 2305-2312.

6. During A, Harrison EH (2007) Mechanisms of provitamin A (carotenoid) and vitamin A (retinol) transport into and out of intestinal Caco-2 cells. J Lipid Res 48: 2283-2294.

7. D'Ambrosio DN, Clugston RD, Blaner WS (2011) Vitamin A metabolism: an update. Nutrients 3: 63-103.

8. Out R, Kruijt JK, Rensen PCN, Hildebrand RB, de Vos P, et al. (2004) Scavenger receptor BI plays a role in facilitating chylomicron metabolism. Journal of Biological Chemistry 279: 18401-18406.

9. N'Soukpoe-Kossi CN, Sedaghat-Herati R, Ragi C, Hotchandani S, Tajmir-Riahi HA (2007) Retinol and retinoic acid bind human serum albumin: stability and structural features. Int J Biol Macromol 40: 484-490.

10. Hogarth CA, Griswold MD (2010) The key role of vitamin A in spermatogenesis. J Clin Invest 120: 956-962.

11. Sugimoto R, Nabeshima Y, Yoshida S (2012) Retinoic acid metabolism links the periodical differentiation of germ cells with the cycle of Sertoli cells in mouse seminiferous epithelium. Mech Dev 128: 610-624.

12. Imanishi Y, Sun W, Maeda T, Maeda A, Palczewski K (2008) Retinyl Ester Homeostasis in the Adipose Differentiation-related Protein-deficient Retina. J Biol Chem 283: 25091-25102.

13. Lillis AP, Van Duyn LB, Murphy-Ullrich JE, Strickland DK (2008) LDL receptor-related protein 1: unique tissue-specific functions revealed by selective gene knockout studies. Physiol Rev 88: 887-918.

14. Lane MA, Chen AC, Roman SD, Derguini F, Gudas LJ (1999) Removal of LIF (leukemia inhibitory factor) results in increased vitamin A (retinol) metabolism to 4-oxoretinol in embryonic stem cells. Proc Natl Acad Sci U S A 96: 13524-13529.

15. Quadro L, Hamberger L, Colantuoni V, Gottesman ME, Blaner WS (2003) Understanding the physiological role of retinol-binding protein in vitamin A metabolism using transgenic and knockout mouse models. Mol Aspects Med 24: 421-430.
